# Metabolomics of Rice Bran Differentially Impacted by Fermentation With Six Probiotics Demonstrates Key Nutrient Changes for Enhancing Gut Health

**DOI:** 10.3389/fnut.2021.795334

**Published:** 2022-02-16

**Authors:** Yohannes Seyoum, Christèle Humblot, Bridget A. Baxter, Nora Jean Nealon, Annika M. Weber, Elizabeth P. Ryan

**Affiliations:** ^1^Center for Food Science and Nutrition, College of Natural and Computational Sciences, Addis Ababa University, Addis Ababa, Ethiopia; ^2^QualiSud, Université de Montpellier, Avignon Université, CIRAD, Institut Agro, IRD, Université de la Réunion, Montpellier, France; ^3^Department of Environmental and Radiological Health Sciences, Colorado State University, Fort Collins, CO, United States; ^4^Department of Food Science and Human Nutrition, Colorado State University, Fort Collins, CO, United States

**Keywords:** bacteria, metabolite, nutrition, yeast, fermentation, rice bran, probiotics

## Abstract

The consumption of rice bran has been shown to have a positive effect on nutritional status and prevention of chronic diseases related to hundreds of metabolites with bioactivity. Consumption after fermentation can lead to specific beneficial effects, yet is lacking complete characterization when fermented with diverse strains. The objective of this study was to examine the effect of fermentation on the rice bran metabolite profile. Bacterial probiotics (*Bifidobacterium longum, Limosilactobacillus fermentum, Lacticaseibacillus paracasei, Lacticaseibacillus rhamnosus* and, *Escherichia coli*) were used to ferment rice bran alone or after incubation with yeast probiotic *Saccharomyces boulardii*. Fermented rice bran was methanol extracted and analyzed by UPLC-MS/MS. The metabolome of the two fermentation types was deeply modified when compared with non-fermented rice bran. The two-step fermentation provided alternative substrate to the bacteria in a few cases. Key metabolites of high nutritional value (essential amino acids, vitamins) and gut health (arabinose, maltotriose) were identified.

## Introduction

The outer layer of rice grain, rice bran, has a unique profile, as it is rich in different nutrients and phytochemicals (1). Rice bran is available everywhere where rice is produced and can be used as a lever to prevent different disorders. Indeed, rice bran, and some of its bioactive compounds, has been shown to have many benefits including prevention of colon cancer and diarrheal pathogens (2, 3). Before rice bran is consumed, it must be heat-stabilized to inactivate rancidity-inducing enzymes (lipases, lipoxygenases) and thereby increasing its shelf life (4). Heat-stabilized rice bran still contains a variety of bioactive compounds that span multiple chemical classes (5).

The hundreds of metabolites in heat-stabilized rice bran account for its positive effects on nutritional status and on the prevention of chronic diseases. Metabolites found in rice bran such as Vitamin E components and Vitamin B6 isoforms were increased in infants consuming rice bran (6). Overall, the metabolomic changes improved infant and child nutritional status and metabolites identified were related with a healthy immune system (6, 7). Many changes also occurred in the gut microbiota compartment since it has been shown that regular consumption of rice bran beneficially shaped the fecal microbiota of infants in Nicaragua and Mali, and it also improved growth scores and gut permeability of infants (6).

The human gut microbiota contains 10^14^ bacteria that provide a diverse range of metabolites and metabolic activities to complement host physiology (8, 9). The role of gut microbiota in health is now widely recognized, and has been linked with preventing the proliferation of pathogens, development of obesity and cardiovascular disease, and plays a role in vitamin synthesis (9). As a consequence, changes in the gut microbiota may affect health. A pilot study in healthy adults showed an increase in the proportion of the *Bifidobacterium longum* after consuming 30 g/day of heat-stabilized rice bran for 28 days. These findings suggested colonic fermentation of rice bran by *Bifidobacterium longum* may elicit health protective effects (7). *Bifidobacterium* are known to improve barrier functions or cross-feeding of other gut bacterial members through the production of acetate that promotes the growth of other beneficial microbial species (10). The metabolomes in stool samples from infants fed rice bran exhibited a number of modifications (6). For example, increased levels of amino acids such as glycine and cysteine were observed and were attributed to a change in gut microbiota metabolism (6).

Rice bran consumption was associated with a number of beneficial of effects, which can be further increased by fermentation. Fermentation has been used for thousands of years in the preservation of raw foodstuffs, it improves not only the organoleptic and sanitary quality of food, but also its nutritional composition. For example, improved protein digestibility has been reported (11) as well as improvement of the amino acid profile (12). The probiotic properties of many microorganisms have also been characterized, and a number of bacteria and yeast (e.g., *Saccharomyces boulardii*) have been used as dietary supplements for the prevention and treatment of intestinal diseases (13). Probiotics are live beneficial organisms, that when consumed in adequate amounts, confer health benefits to the host (14). Probiotic species found naturally in the guts of people and animals include *Bifidobacterium longum, Escherichia coli, Limosilactobacillus fermentum, Lacticaseibacillus paracasei* and *Lacticaseibacillus rhamnosus*. Previous work using the same strains than the present study have already been used to ferment rice bran, and different positive effects have been shown for suppressing the growth of *Salmonella*, protecting against human rotavirus diarrhea, and increasing the concentration of colorectal cancer protective metabolites in the colon tissue and blood (3, 15, 16). Fermentation of rice bran with *Saccharomyces cerevisiae* var. *boulardii* ATCC MYA-797 (*S. boulardii*) produced novel metabolite profiles with bioactivity (17). *L. paracasei* ATCC 27092 fermentation of rice bran reduced the growth of *Salmonella typhimurium in vitro*. This was linked with major modifications of the metabolic profile of rice bran after fermentation (16). In an *in vivo* study in mice, mice fed rice bran fermented with *B. longum* ATCC 55,813 as compared to mice fed non-fermented rice bran fed, had profound changes of their gut microbiota (15). Furthermore, pigs fed a combination of *L. rhamnosus* GG ATCC 553103 and *E. coli* Nissle 1,917 together with rice bran were protected against human rotavirus induced diarrhea (3). Thus, the profound changes in its metabolite profile brought about by fermentation of rice bran may have beneficial consequences for health after ingestion.

While the health benefits of fermented rice bran have been highlighted here, no previous study has been conducted on the specific metabolomic changes related to the fermentation of rice bran both by a one-step fermentation by probiotic bacteria and by a two-step yeast and bacterial fermentation. The hypothesis is that the fermentation with the probiotic yeast provides alternative substrates for the production of specific metabolites by the probiotic bacteria. Therefore, the objective of this work was to examine the contributions of six probiotic microorganisms to produce rice bran microbial metabolites of importance for nutrition and intestinal health. Each probiotic was used alone (one-step fermentation) or each bacterial probiotic was used following a rice bran fermentation with the yeast probiotic *S. boulardii* (two-steps fermentation). We hypothesized that bacteria and yeast used in this study will improve nutrient profile in rice bran as well as reduce anti-nutrients.

## Materials and Methods

### Rice Bran

The Rice Bran Technologies-300 (RBT-300) is a commercial rice bran used in this study (California, USA). As described previously, RBT-300 rice bran is heat-stabilized and milled and was stored after purchase at 4°C until use (18).

### Probiotic Microorganisms Used for Fermentation

Four probiotic strains (*B. longum* ATCC 55813, *L. fermentum* ATCC 23271, *L. paracasei* ATCC 21052 and *L. rhamnosus* GG ATCC 53103) were used in this study and obtained from American Type Culture Collection (ATCC), Manassas, VA, USA. *E. coli* Nissle 1917 and *L. rhamnosus* GG were donated by Dr. Lijuan Yuan at the Virginia Polytechnic Institute. *Saccaromyces cerevisia*; Sb49 boulardii (ATCC MYA-797) was purchased from ATCC (Manassas, VA, USA).

### Rice Bran Fermentation

Rice bran was fermented in one step (bacteria or yeast alone) or in two steps (yeast followed by bacteria). The isolates of *L. fermentum, L. paracasei, L. rhamnosus and E. coli* were maintained in de Man, Rogosa and Sharpe (MRS) broth with 20% glycerol and stored at −80°C. The *B. longum* was maintained in Bifidus Selective Medium (BSM) broth containing 20% glycerol and stored at −80°C. *S. boulardii* reactivated from stock cultures maintained in yeast nitrogen base (YNB) amended with 0.5% ammonium sulfate and 2% dextrose (stored at −80°C in 20% glycerol). The stock cultures for all yeast and bacteria were sub-cultured once in fresh, sterile YNB, MRS or BSM broth before being used for fermentation under aerobic conditions at 37°C. All probiotics were incubated at 37°C, for 24 h (*Lactobacilli* and *E. coli* Nissle) or 48 h (*B. longum* and *S. boulardii*).

The resultant cultures were washed in phosphate buffer solution and resuspended in water to obtain a suspension containing 6 × 10^5^ CFU/mL. All fermentation were done in in a portable 19 L fermentation container designed to mimic microaerophilic conditions along the periphery and anaerobic conditions in the center of the fermentation vat. In the one-step fermentation, 500 mL of each probiotic bacterial suspension was added to 1 kg of heat stabilized rice bran batch and incubated for 48 h at room temperature. For fermentation with *S. boulardii*, the inoculum was prepared in a similar way to the bacteria, with 6 × 10^5^ CFU/mL of the *S. boulardii* suspension was added to 1 kg of heat stabilized rice bran and left to ferment for 24 h. In the two-step fermentation, bacteria were inoculated using the same process on rice bran previously fermented with *S. boulardii*. Following fermentation, fermented rice bran was lyophilized and stored at 4°C until being processed. All fermentations were carried out in triplicate.

### Metabolite Extraction

Fermented rice bran was sent to Metabolon Inc^©^ (Durham NC, USA) for metabolite extraction and metabolite identification using previously established protocols. Metabolites were extracted from fermented rice bran with 80% methanol under vigorous shaking for 2 min (Glen Mills GenoGrinder 2,000) followed by centrifugation to remove proteins, dissociate small molecules bound to protein or trapped in the precipitated protein matrix, and to recover chemically diverse metabolites. This extraction method has been previously shown to allow for the resolution and identification of metabolites with a wide range of hydrophobicities (long-chain lipids, sterols to phytochemicals with large hydrophilic moieties) which belong to many of the rice bran bioactives we are interested in (1, 17). The resulting extract was divided into five fractions for chromatographic extraction including two separate reverse-phase (RP) ultra-high performance liquid chromatography (UPLC) tandem mass-spectrometry (UPLC-MS/MS) with positive ion mode electrospray ionization (ESI), one for UPLC-MS/MS with negative ion mode ESI, one for hydrophilic-interaction (HILIC)/UPLC-MS/MS with negative ion mode ESI and one backup sample. Samples were placed briefly on a TurboVap^®^ (Zymark) to remove the organic solvent. The sample extracts were stored overnight under nitrogen before preparation for analysis.

### Non-targeted Metabolomics

Metabolite profiling was performed using a Waters ACQUITY UPLC and a Thermo Scientific Q-Exactive high resolution/accurate mass spectrometer interfaced with a heated electrospray ionization (HESI-II) source and an Orbitrap mass analyzer operated at 35,000 mass resolution (Waltham, MA, USA). The sample extract was dried then reconstituted in solvents compatible with each of the four methods. Each reconstitution solvent contained a series of standards at fixed concentrations to ensure injection and chromatographic consistency.

One aliquot was analyzed using acidic positive ion conditions, chromatographically optimized for more hydrophilic compounds. In this method, the extract was gradient eluted from a C18 column (Waters UPLC BEH C18-2.1 × 100 mm, 1.7 μm) using water and methanol, containing 0.05% perfluoropentanoic acid (PFPA) and 0.1% formic acid (FA). Another aliquot was analyzed using acidic positive ion conditions, but was chromatographically optimized for more hydrophobic compounds. In this method, the extract was gradient eluted from the same aforementioned C18 column using methanol, acetonitrile, water, 0.05% PFPA and 0.01% FA and was operated at an overall higher organic content. Another aliquot was analyzed using basic negative ion optimized conditions using a separate dedicated C18 column. The basic extracts were gradient eluted from the column-using methanol and water with a 6.5 mM Ammonium Bicarbonate mobile phase at pH 8. The fourth aliquot was analyzed via negative ionization following elution from a HILIC column (Waters UPLC BEH Amide 2.1 × 150 mm, 1.7 μm) using a gradient consisting of water and acetonitrile with 10 mM Ammonium Formate, pH 10.8. The MS analysis alternated between MS and data-dependent MSn scans using dynamic exclusion. The scan range varied slighted between methods but covered 70–1,000 m/z.

Several controls were analyzed along with each experimental sample. Samples were spiked with a cocktail of known chemical standards before UPLC-MS/MS. A pooled matrix of samples containing an equal volume of each experimental samples were analyzed to control for sample drift across successive runs, and extracted water samples were used as process blanks. Compounds were identified based on an internal Metabolon library containing over 3,300 commercially available chemical standards. Metabolite annotations were based on matches with the retention time/index, where a positive match was defined as having an m/z within 10 ppm to a database standard, and by matching the overall mass spectral profile with database standards using both forward and reverse spectral scores. Metabolites that were identified by in-silico algorithms vs. a chemical standard were annotated with “^*^” (Supplementary Table 1). Spectral profiles that were structurally resolved but were otherwise not archived in internal chemical database are reported as “unknown”.

### Statistical Analysis

Metabolite raw abundances were normalized by dividing the median raw abundance of that metabolite across the entire dataset for each matrix, resulting in median scaled abundances. For samples lacking a metabolite, the minimum median scaled abundance of that metabolite across the dataset was imputed as a minimum value before downstream statistical analysis. Metabolite fold differences were calculated for each metabolite by dividing the average median scaled abundance of the metabolite in one treatment group by that of a second treatment group. All samples were run in technical triplicates to approximate a Gaussian distribution. Prior to principal components analysis (PCA), all metabolites from this experiment were median-scaled for the metabolite value across the dataset for normalization. This method of normalization allows all metabolites in the dataset to be compared as equivalent terms to each other. Analysis by two-way ANOVA identified biochemicals exhibiting significant differences for experimental parameters. Interactions and main effects for experimental parameters of probiotic bacteria and probiotic yeast, where significance was defined as *P* < 0.05. To account for false discovery rate errors, a q-value was calculated for each metabolite and metabolites with a value of *q* < 0.1 were excluded from downstream analysis.

## Results

### Modulation of the Rice Bran Metabolome by One- and Two-Step Probiotic Fermentation

**Figure 1:**
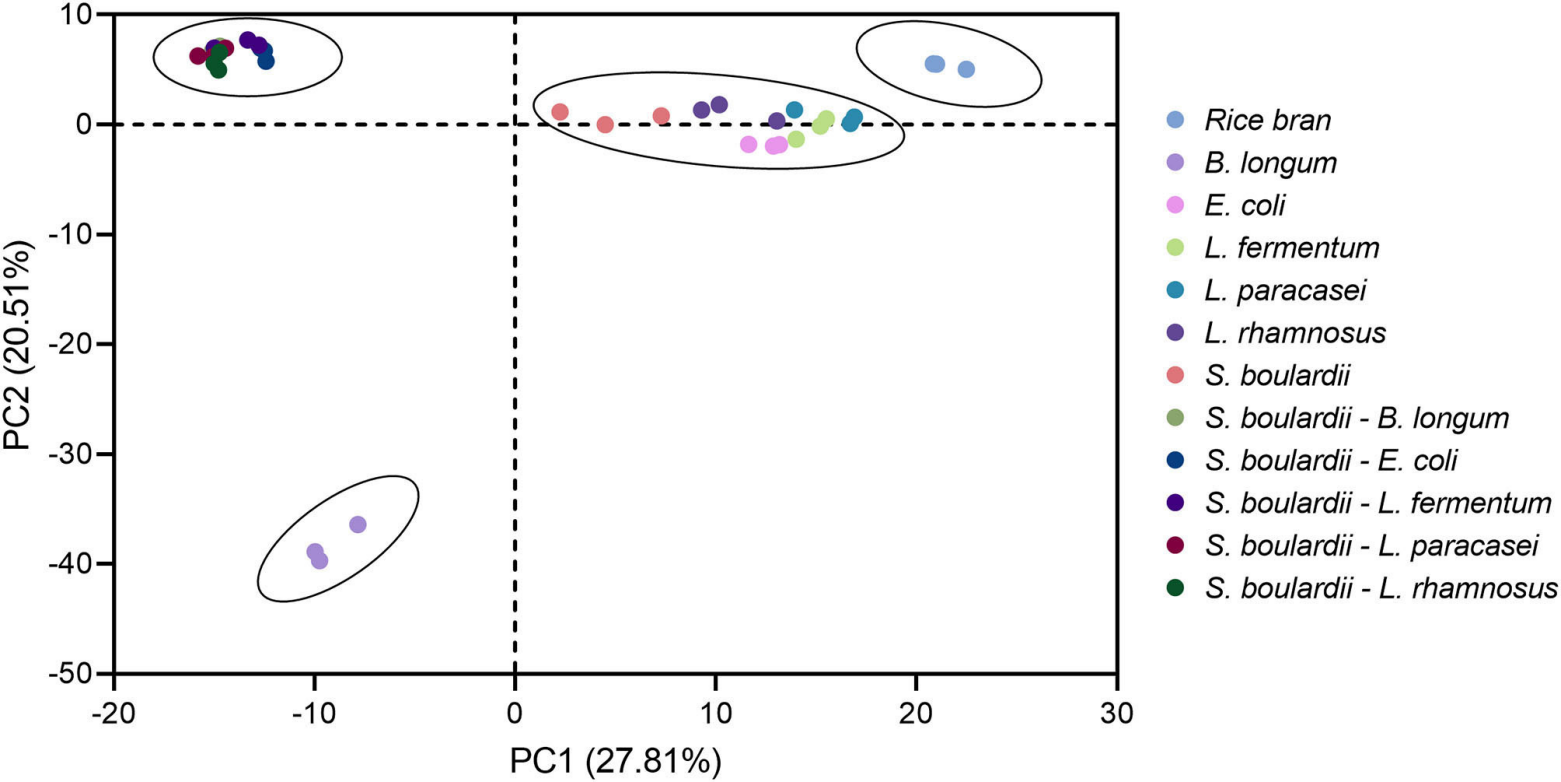
Principal component analysis depicts the metabolome clusters and separations between rice bran alone and the bacteria or yeast+bacteria fermentation of rice bran. All metabolites from this experiment were median-scaled for the metabolite value across the dataset for normalization.

The rice bran metabolite profile in the all fermentation conditions yielded 613 unique biochemical compounds of known identity across 8 major chemical classes. The results of principal component analysis of the metabolomic profiles of rice bran in all 11 fermentation conditions are shown in Figure 1. The PCA model shows 27.81% variance for PC1 and 20.51% for PC2. As expected, all the fermented rice bran metabolite profiles were separated from non-fermented control rice bran. One-step fermentation with *E. coli, L. fermentum, L. plantarum* and *L. rhamnosus* were closer compared to one-step fermentation with *B. longum*. The two-step fermentation profiles all segregated together.

### Rice Bran Metabolites Displayed Differences Across Metabolic Pathways and Were Dependent on the Probiotic Used for Fermentation

The number and types of rice bran compounds changed by the one-step and two-step fermentation protocol were analyzed according to classifications as amino acid, peptide, carbohydrate, lipid, cofactors and vitamins, nucleotide, phytochemicals, and partially characterized molecules.

**Figure 2:**
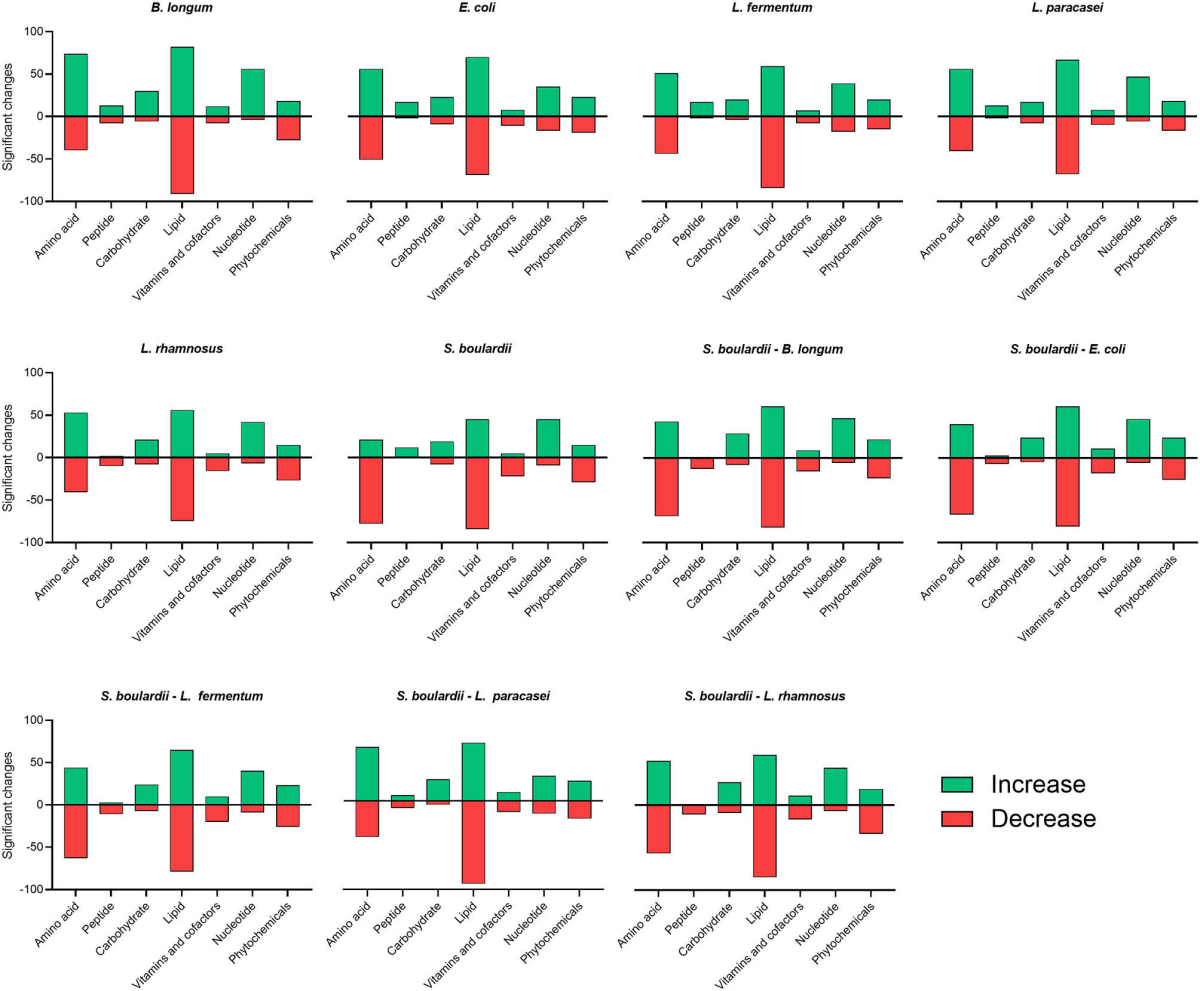
Significant changes in the number of metabolites from distinct chemical groups across all fermentation conditions were compared with non-fermented rice bran. Amino acid, peptide, carbohydrate, lipid, nucleotide, cofactors and vitamin and phytochemicals classifications were impacted by fermentation. The number of metabolites that increased after fermentation is shown in green, the number of metabolites that decreased after fermentation is shown in red.

Figure 2 shows the number of metabolites analyzed across 11 distinct fermentation conditions and which exhibited significant differences from non-fermented rice bran. Seventy four metabolites involved in amino acid metabolism increased (*P* < 0.05) when rice bran was fermented with *B. longum* compared with all the other fermentations, particularly with *S. boulardii* only 21 metabolites were increased. Metabolites involved in peptide metabolism were the most contrasted depending on the probiotic used for fermentation. They mainly decreased (*P* < 0.05) in one-step fermentations with *L. rhamnosus* (10 metabolites) and in all two-steps fermentation, and mainly increased in all other conditions. Metabolites related to nucleotide metabolism increased (*P* < 0.05) in most cases, but some decreased when rice bran was fermented with *E. coli* and *L. fermentum* alone. Fermentations involving yeast alone or combined with probiotic bacteria led to a decrease in many of the metabolites involved in co-factor and vitamin metabolism. This was also the case of rice bran fermented with *L. rhamnosus* alone. The number of metabolites involved in carbohydrate, lipid and phytochemicals chemical classes were similarly affected by all fermentation conditions, but the metabolites in each category were affected differently.

### Rice Bran Fermentation With the Different Probiotics Increased Vitamin Levels

**Figure 3:**
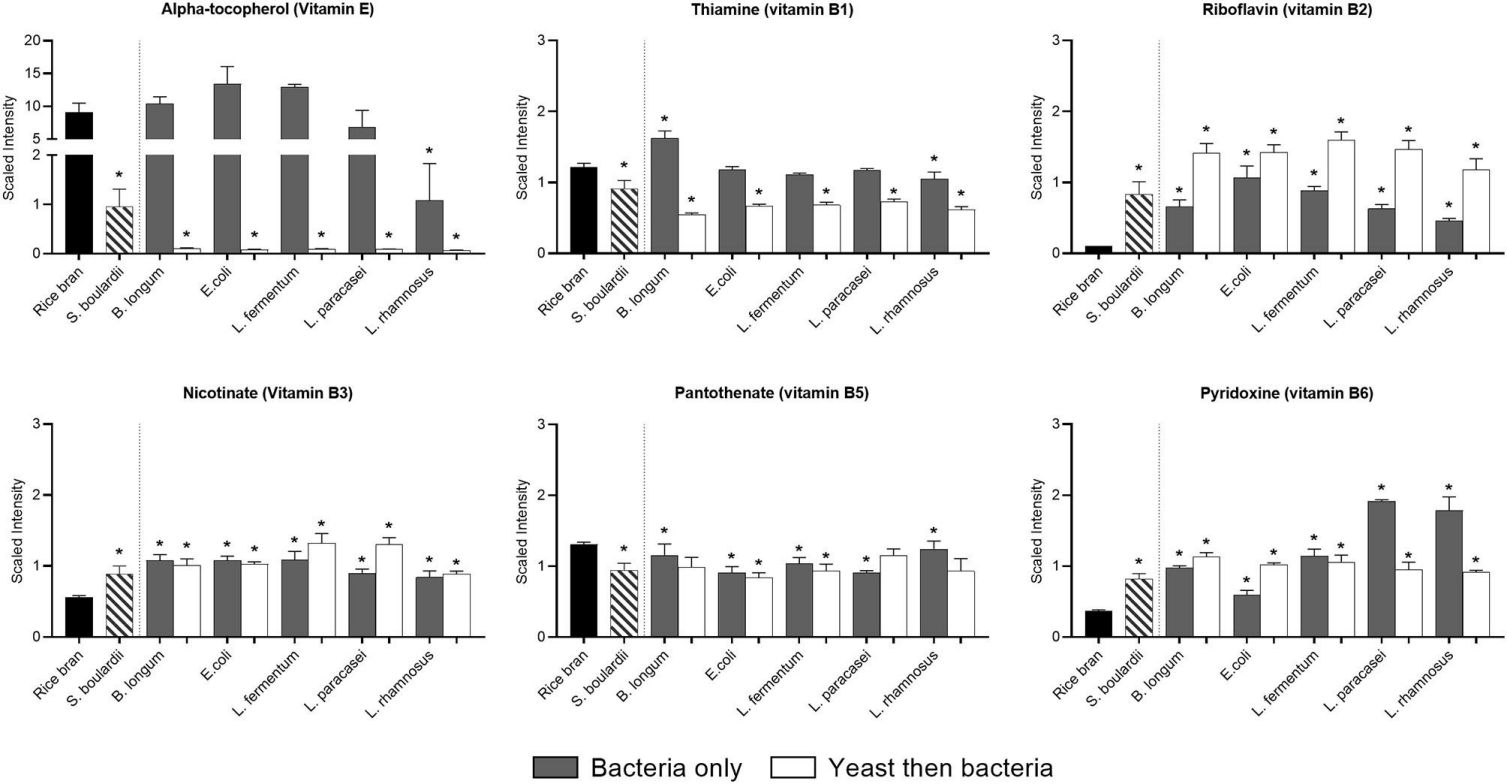
Median-scaled relative abundance changes for vitamins found in rice bran before and after fermentation using bacteria or yeast alone or yeast followed by bacteria. ^*^ indicate significant differences between the fermentations when compared to the relative abundance in non-fermented rice bran. Significance determined by 2-way ANOVA (*p* < 0.05).

All fermentations affected the vitamin profile of rice bran (Figure 3). For instance, alpha-tocopherol was substantially decreased by fermentation using *L. rhamnosus* (0.88-fold) and *S. boulardii* alone (0.9-fold), or when *S. boulardii* fermentation was followed by fermentation in all bacterial strains (*P* < 0.05). Ascorbate relative abundance remained stable or was slightly reduced (*P* < 0.05; ~0.4-fold) after one-step (*L. paracasei* and *L. rhamnosus*) and two-step fermentation (*E. coli, L. fermentum* and *L. rhamnosus*).

The vitamin B profile was drastically modified by all fermentation conditions. The relative proportion of pantothenate (vitamin B5) was negatively affected with only a slight decrease (~0.25-fold) in most of the fermentations (*P* < 0.05). Conversely, all fermentation conditions increased (*P* < 0.05) the relative abundance of pyridoxine (vitamin B6) and riboflavin (vitamin B2), with a marked effect of some probiotics [i.e., *L paracasei* (4.3-fold) and *L. rhamnosus* (3.9-fold) for pyridoxine and all two-step fermentations for riboflavin (up to 14.7-fold)]. The relative abundance of thiamin (vitamin B1) only increased (0.3-fold) when *B. longum* was used alone for fermentation, whereas it decreased with all two-step fermentations (0.2–0.5-fold; *P* < 0.05). Different forms coexisted within one vitamin, and these were differentially affected by fermentation. This was the case of vitamin B3, where nicotinamide drastically decreased in all fermentations (up to 1-fold), whereas nicotinate increased in all fermentation (0.6–1.4-fold; *P* < 0.05). The metabolomics approach also allowed us to see the precursors and consequent metabolites that decreased or increased depending on the proportion of the vitamin (Supplementary Table 1).

### Amino Acid Profile Alterations After Fermentation With the Different Probiotic Strains

**Figure 4:**
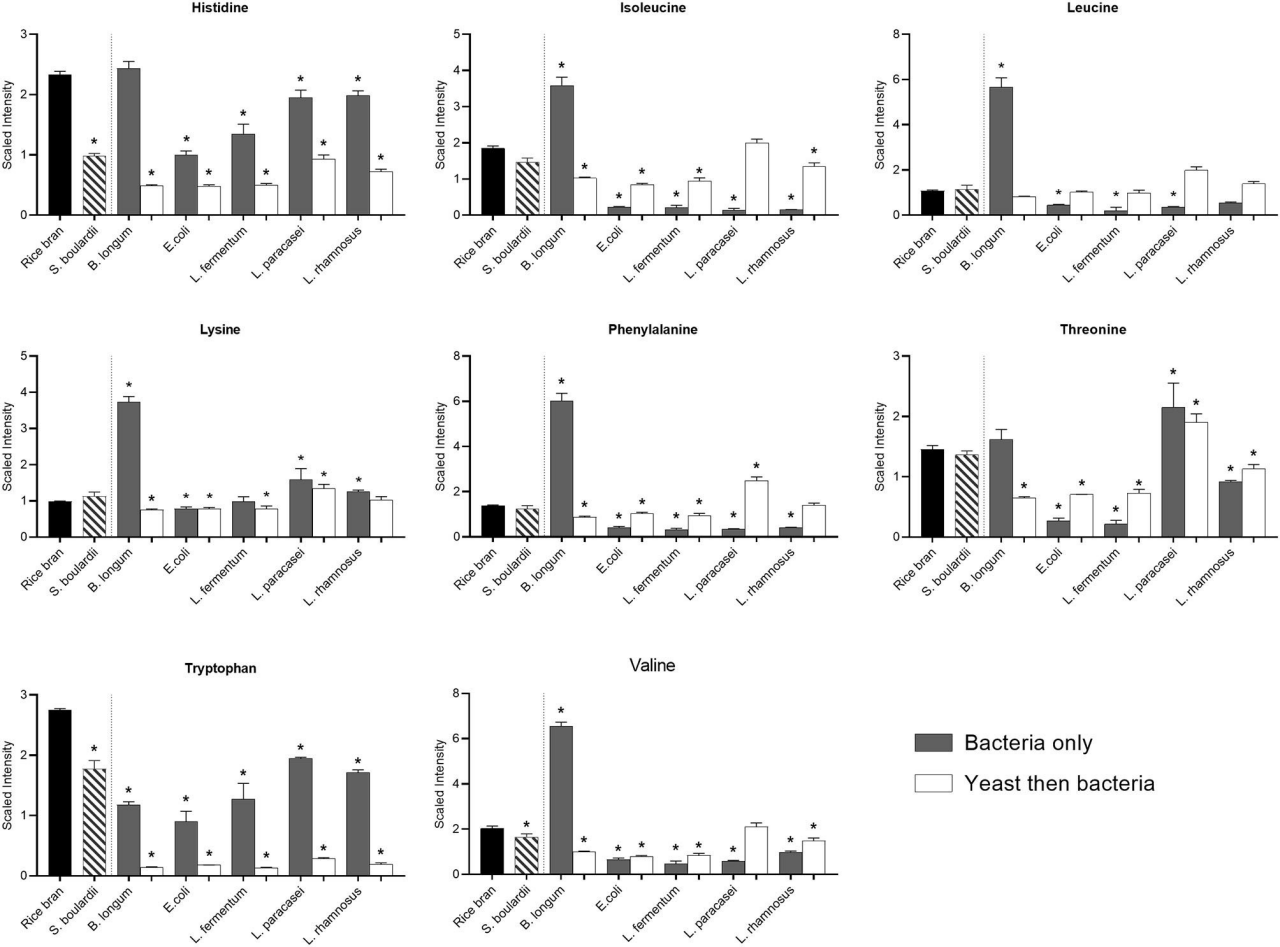
Median-scaled relative abundance changes for essential amino acids found in rice bran before and after fermentation using bacteria or yeast alone or yeast followed by bacteria. ^*^ are noted for metabolites with significant differences identified between non-fermented rice bran across the fermentation conditions as analyzed by ANOVA (*p* < 0.05).

Fermentation affected the essential amino acid profiles differently depending on the strain of bacteria and yeast, and/or their combinations. The bacteria that modified most of the essential amino acid profiles of rice bran was *B. longum* (Figure 4). After *B. longum* fermentation, there was a significant increase in the concentration of isoleucine (0.9-fold), leucine (4.3-fold), lysine (2.8-fold), phenylalanine (3.4-fold), and valine (2.2-fold) (*P* < 0.05). However, when *B. longum* used in combination with *S. boulardii*, the relative abundance of all essential amino acids were decreased (*P* < 0.05), the exception being leucine. In addition, the other bacteria used alone or after *S. boulardii* fermentation also modified the essential amino acid profile but mainly by reducing their concentration. The exception was threonine, whose level increased when *L. paracasei* (0.5-fold) was used alone or combined with *S. boulardii* (0.3-fold) for fermentation (*P* < 0.05).

### Carbohydrate Profile of Rice Bran Fermented With the Different Probiotics

**Figure 5:**
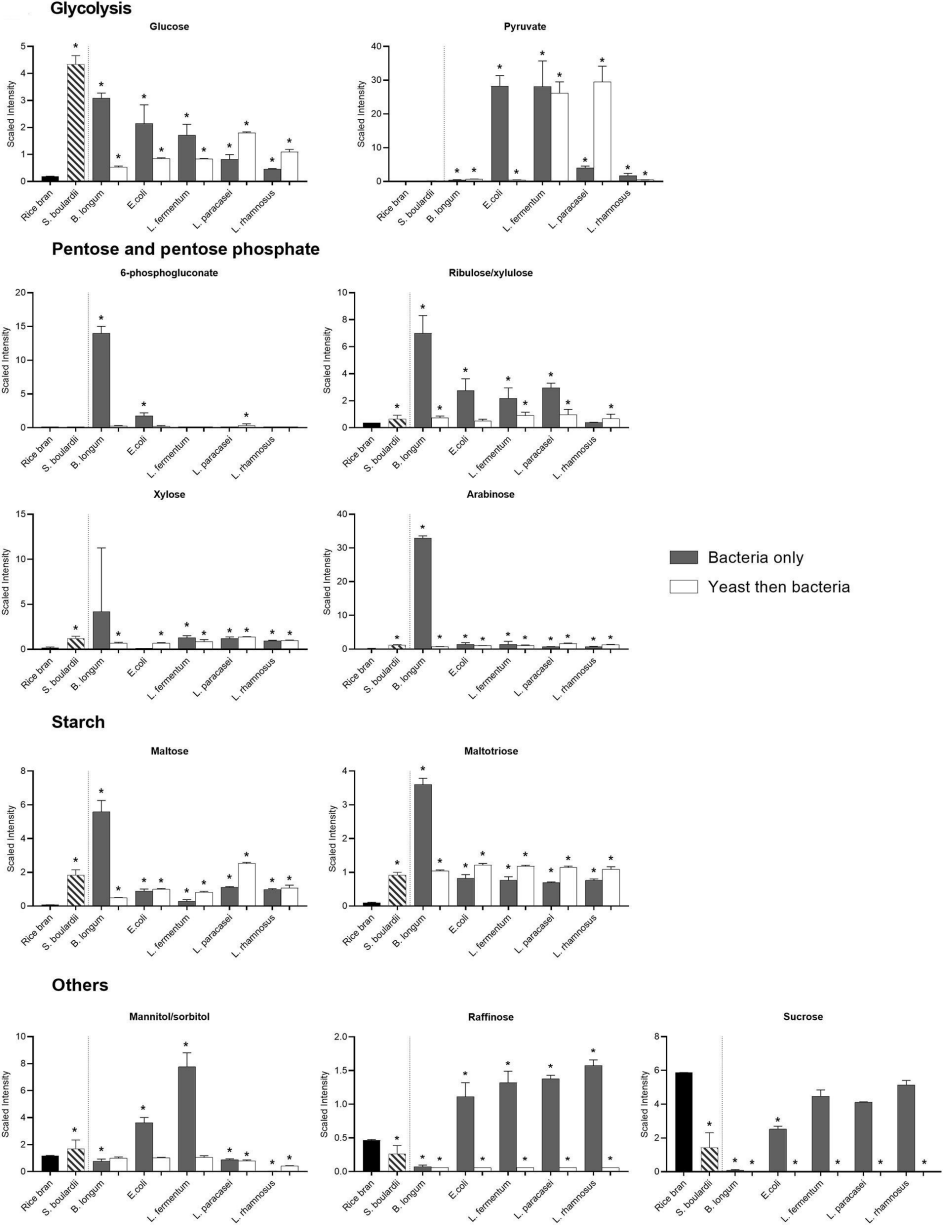
Median-scaled relative abundance showed as scaled intensity for a selection of rice bran carbohydrates before and after fermentation using bacteria or yeast alone or yeast followed by bacteria. ^*^ are noted for significant differences identified between non-fermented rice bran across the fermentation conditions as analyzed by ANOVA (*p* < 0.05).

The carbohydrate pathways preferentially used by the different strains were revealed by the metabolites identified after fermentation (Figure 5). For example, the metabolism of pentose was low in most of the bacterial probiotics with the exception of *B. longum*. Indeed, the relative abundance of 6-phosphogluconate (91-fold) and arabinose (181-fold) increased mainly in rice bran fermented with *B. longum* (*P* < 0.05). The relative abundance of ribulose was also higher with *B. longum* (17.8-fold) even if increases were also observed with *E. coli* (6.5-fold), *L. fermentum* (5-fold), *L. paracasei* (7-fold) in one-step fermentation (*P* < 0.05). Conversely, all the metabolites in the glycolysis pathway (glucose, glucose-6-phospate, fructose-1,6-diphosphate, 2-phosphoglycerate, 3-phosphoglycerate, polyenolpyruvate and pyruvate) were detected, although at different intensities depending on the fermentation conditions (Supplementary Table 1; Figure 5).

Starch metabolism was revealed by the increase in the relative abundance of maltose and maltotriose (Figure 5). All fermentations increased the relative abundance of theses metabolites slightly, but again, the most pronounced effect was observed with *B. longum* (~70-fold; *P* < 0.05). Metabolism of other simple sugars also led to an increase in the metabolites depending on the probiotic used for fermentation. For example, the relative abundance of mannitol increased considerably with *E. coli* (2-fold) and *L. fermentum* (5.6-fold), whereas its relative abundance was less affected by all the other fermentations (Figure 5).

The sucrose initially present in the rice bran was almost totally consumed by all the bacteria in the two-step fermentation, and by *S. boulardii* and *E.coli*, when used for fermentation alone. As expected, lactate production was higher in fermentation involving lactic acid bacteria (Figure 5).

If prebiotics can support the growth of probiotics, some specific metabolites belonging to the prebiotic families can be synthesized by microorganisms. Indeed, the relative abundance of raffinose slightly increased (1.3–2.3-fold) with all bacteria except *B. longum* (0.84-fold) in the one-step fermentation (*P* < 0.05). *B. longum* specifically produced xylose (21-fold) and arabinose (181-fold), which can also have prebiotic effects. Interestingly, the high amount of xylose and arabinose observed after fermentation with *B. longum* alone may reflect higher content in arabinoxylan, constituted of these two polysaccharide fractions.

### Relative Abundance of the Chemical Class of Lipids in Rice Bran Fermented or Not With the Different Combinations

**Figure 6:**
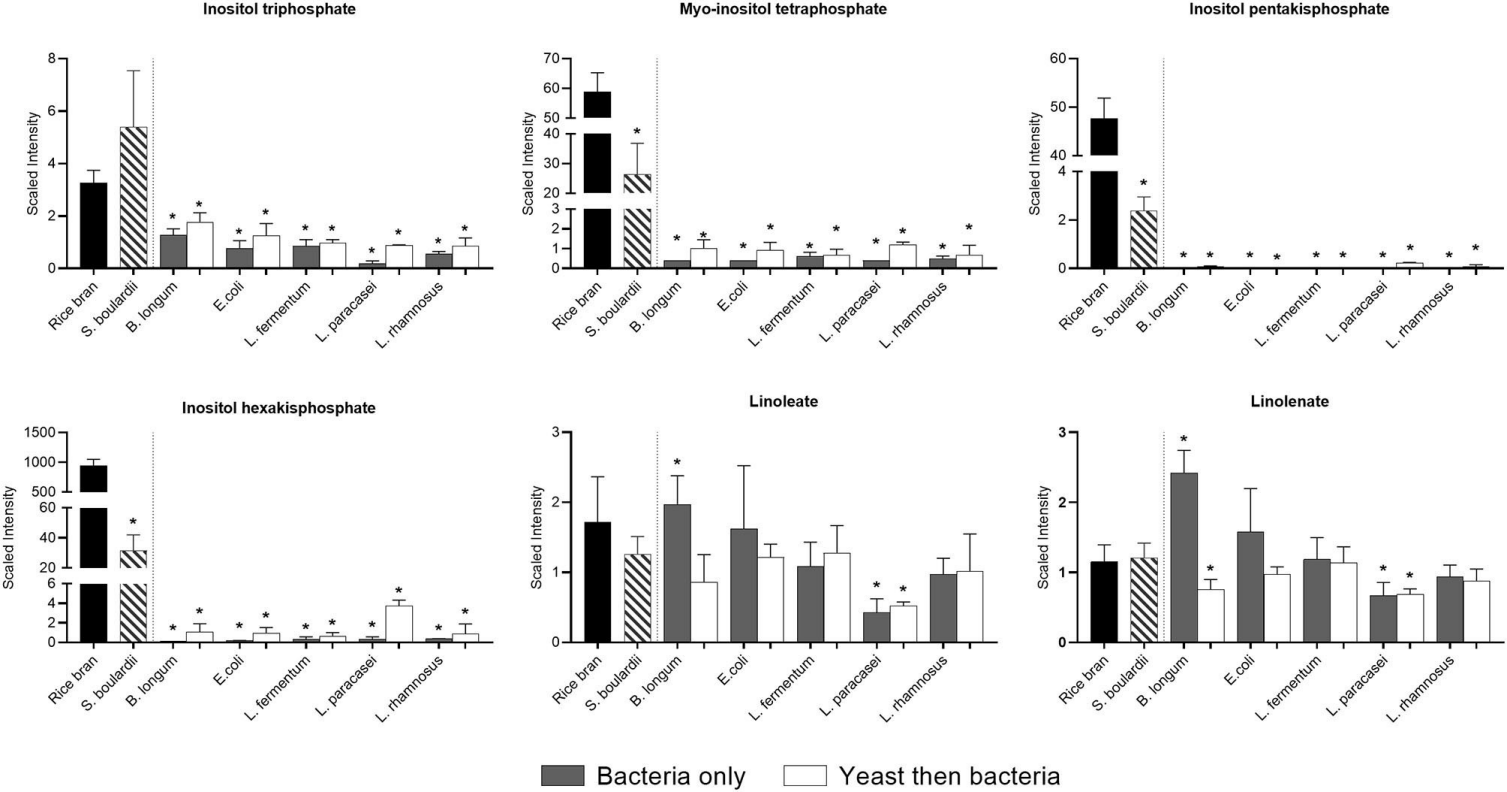
Median-scaled relative abundance showed as scaled intensity for a selection of rice bran lipids across fermentation treatments. Significant differences were identified in rice bran lipids before and after fermentation using bacteria or yeast alone or yeast followed by bacteria. ^*^ are noted for significant differences identified between non-fermented rice bran across the fermentation conditions as analyzed by ANOVA (*p* < 0.05).

Figure 6 shows that the relative abundance of essential fatty acids such as linoleate, did not change remarkably due to fermentation, with the exception of linolenate, which increased slightly when *B. longum* was used for fermentation alone. Interestingly, inositol tri-tetra and hexaphosphates that likely originated from the rice bran were almost completely degraded during fermentation by all bacteria used alone or after yeast fermentation.

### Relative Abundance of Other Bioactive Components

The relative abundance of different plant components was also modified by fermentation (Supplementary Table 1). For example, the relative abundance of vanillin decreased in all fermentation conditions (0.5–0.8-fold) except with *L. rhamnosus* (increased 0.15-fold). Vanillate decreased during fermentation with *B. longum* (0.5-fold, *P* < 0.05) and *L. rhamnosus* (0.2-fold, *P* < 0.05) alone, however it increased in all two-step fermentations (0.5–1-fold, *P* < 0.05). The relative abundance of ferulate increased during fermentation with all strains (1–3.2-fold, *P* < 0.05) except *B. longum* (0.3-fold, *P* < 0.05) and *L. rhamnosus* (0.8-fold, *P* < 0.05).

## Discussion

The fermentation of rice bran with the different probiotic microorganisms resulted in a dramatic modification of the rice bran metabolome in one-step as well as in two-step fermentations. Some metabolites of importance to nutrition (e.g., vitamins and essential amino acids) and gut health (e.g., prebiotics) were produced, and some anti-nutritional factors (e.g., phytates) were reduced.

Rice bran is rich in different vitamins including thiamin, niacin, and pyridoxine and we estimated to which extent it can be used to fight micronutrient deficiency. Based on a daily consumption of 5 g per day (nutrient composition as provided by USDA national nutrient database) and recommended nutrient intakes (RNI) for infants (6–12 months), we calculated that even if a contribution is possible, none of the vitamin identified by metabolomics could completely cover the needs (19). In contrast, after fermentation, the marked increase in the relative abundance of vitamin B6 (RNI = 68 mg/day) would cover 110–300% of the RNI, depending on the type of fermentation. Another example was the production of riboflavin, whose concentration is usually very low (2.8 μg/g) in rice bran, but fermentation can increase this concentration up to 15 fold, and the increase in relative abundance is most pronounced with the two-step fermentation, allowing a maximum 50% coverage of nutritional requirements (19). On the contrary, other vitamins, such as niacin, were no longer detected after fermentation. Although yeast are known for the ability to synthesize some B vitamins such as riboflavin, for bacteria this capacity is strain-dependent (20). Indeed, many lactic acid bacteria required riboflavin to grow while others can synthesize it (20). As the fermentation types and conditions may affect the vitamin profile differently, a balance is needed when targeting the population for supplementation (i.e., adults, young children). Indeed, vitamin E is also important to limit the development of non-communicable diseases such as cancer or cardiovascular diseases, which are more frequent in adults. Vitamin E content of rice bran as a composite of tocopherols and tocotrienols is considerably high, though variable across varieties (21). The relative abundance of vitamin E isoforms was less affected by one-step fermentation. When the yeast is used alone, there is a partial consumption of vitamin E, which is most probably consumed by each of the bacteria in the second fermentation step. On the contrary, riboflavin, whose deficiency can lead to anemia, which must be prevented in young children, increased to a greater extent following two-step fermentation.

Some other essential components such as amino acids were also affected by fermentation. Most of the fermentations had no effect or had a negative effect on the essential amino acid profiles. The exception was fermentation with *B. longum* alone, which was able to increase the relative abundance of isoleucine, leucine, lysine, phenylalanine, and valine. An *in silico* analysis supported the presence of genes coding enzymes involved in the biosynthetic pathways on Kyoto Encyclopedia of Genes and Genomes (22), and confirmed the presence of most of the genes involved in essential amino acid synthesis in the genome of strains belonging to *B. longum* species.

Phytates (myo-inositol phosphate family) are often considered antinutritional for chelating minerals, and thus limiting bioavailability in the gastrointestinal tract has been considered (23). Different intestinal bacteria have been shown to degrade phytates due to specific enzyme activities, and this was documented for bacteria such as *Bacteroides thetaiotaomicron* (24). Nevertheless, as iron absorption occurs mainly in the small intestine, elimination of phytates in food is preferable (25). In the present work, we observed the almost complete elimination of different phytates in all fermentations, suggesting improved mineral absorption. Such a result is not surprising since phytase activity have been identified in many bacteria from the same bacterial species used in this study (25). An elimination of phytates in food has been repetitively associated with an improved mineral absorption in different *in vitro* and *in vivo* models (25).

*B. longum* was the microorganism which most clearly modified the metabolic profile of rice bran, especially in a number of nutritionally important metabolites such as vitamin B1, and some essential amino acids. The *Bifidobacterium* genus is a dominant component of infant gut microbiota, and the ratio of bacteria from this genus has been shown to increase in healthy adults following rice bran supplementation (7, 26). The species of this genus are known for their major contribution to infant metabolism through the degradation of different carbohydrates (26). The bacteria belonging to the genus *Bifidobacterium* are known to contain a range of extracellular enzymes, which enable access to polysaccharides that are too large to be absorbed (27). Indeed, it was estimated that 10% of the total glycosyl hydrolases is located extracellularly and include pullulanases, α-amylases, β-xylosidases, α-L-arabinofuranosidases and α-L-arabinofuranosidases (27). In our study, *B. longum* was the only bacterium to be able to produce starch degradation metabolites such as maltose and maltotriose, thereby underlining its ability to degrade starch. These carbohydrates can have prebiotic effects on the gut, and influence the microbiota metabolism of other species in the gut (28). Xylose and arabinose are components of arabinoxylan, which belongs to the dietary fibers and are known for several health-related properties of these prebiotic compounds. Arabinoxylan are also linked with ferulic acid, very well-known for its antioxidant activity (29). Fermentation also affected the carbohydrate profile of rice bran. All of the probiotic species used in this study have a gene coding for alpha-galactosidases, which can explain the slight increase in the raffinose observed in this study (22). This reduced the number of metabolites such as sucrose, which was no longer detected after fermentation with *B. longum* and all two-step fermentation. Sucrose is not recommended in the case of insulin resistance, so reducing its concentration is desirable for those with diabetes. On the contrary, many other carbohydrates were released, including xylose and xylitol. Interestingly, these carbohydrates are used as sweetening agents for patients with diabetes. Thus, in addition to modifying health-related metabolites, fermentation may also modify the organoleptic quality of rice bran. Some interesting bioactive compounds were not identified using our analytical method, which is the case for polymers. For example, ß-glucan are present in brans of cereals with a large panel of health-related properties (prebiotic, immunomodulatory, anti-cancer, etc.), result in glucose with our pipeline of metabolomic analysis. Such a central metabolite may result from a number of metabolic pathways, making any general interpretation difficult.

In addition to the synthesis of bioactive compounds such as vitamins or essential amino acids described previously, microorganisms can also liberate metabolites from the food matrix. This is the case for ferulate, a polyphenol known to have many physiological functions including anti-inflammatory, antioxidant, antimicrobial, anticancer, and antidiabetic effects (30). In the plant, ferulate is mainly bound to plant cell wall polymers and must be released in its free from to exert its beneficial effects (30). Some metabolites were detected only in the two-step fermentation, underlining the role of the yeast in providing a substrate for the bacteria. This was the case of tyrosol, a phenolic antioxidant also found in olive oil, whose relative abundance only increased in the two-step fermentation (31). The two-step fermentation was clearly of interest for some metabolites, but in practice, it represents additional cost and work for a limited effect since only 40 compounds out of 600 increased in this situation alone.

Metabolomic analysis also allowed a unique view of the general metabolism of the different microorganisms on the same substrate. For example, *B. longum* mainly orientated its carbohydrate metabolisms to the pentose phosphate pathway and the use of starch. *E. coli* preferentially exhibited metabolites from the glycolysis and galactose metabolism pathways. *L. fermentum* fermentation revealed metabolites from glycolysis, lactic acid fermentation and galactose metabolism. Finally, *L. paracasei* and *L. rhamnosus* fermentation exhibited metabolites from lactic acid fermentation and galactose metabolism pathways.

## Conclusion

Metabolic differences between probiotic fermentations of rice bran performed in this study clearly demonstrated a nutritional advantage for influencing health and organoleptic properties. A judicious choice of bacteria (i.e., *B. longum*) can improve the initial composition of rice bran by enhancing the nutrient composition that has global health applications for preventing nutritional deficiencies through local and sustainable diet-based solutions. Future directions for this work include the nutrient bioavailability assessments following consumption of fermented rice brans.

## Data Availability Statement

The raw data supporting the conclusions of this article will be made available by the authors, without undue reservation.

## Author Contributions

BB, ER, and NN contributed to conception, design of the study, and collected the metabolomics data. AW, CH, ER, and YS performed the statistical analysis. CH and YS wrote the first draft of the manuscript. All authors contributed to the article and approved the submitted version.

## Funding

This research was supported by the National Institutes of Health- Office of Dietary Supplements as an administrative supplement to the National Cancer Institute award R01CA201112-05 to ER. Stipends were provided from the Montpellier University of Scholarly Excellence (MUSE) for ER and from the French Embassy in Ethiopia for YS.

## Conflict of Interest

The authors declare that the research was conducted in the absence of any commercial or financial relationships that could be construed as a potential conflict of interest.

## Publisher's Note

All claims expressed in this article are solely those of the authors and do not necessarily represent those of their affiliated organizations, or those of the publisher, the editors and the reviewers. Any product that may be evaluated in this article, or claim that may be made by its manufacturer, is not guaranteed or endorsed by the publisher.

## Supplementary Material

Supplementary Table 1 Heat map of statistically significant biochemicals profiled in this study.
